# Applications of Deep Eutectic Solvents in Sample Preparation and Extraction of Organic Molecules

**DOI:** 10.3390/molecules27227699

**Published:** 2022-11-09

**Authors:** Orfeas-Evangelos Plastiras, Victoria Samanidou

**Affiliations:** 1 UMR CNRS 8181, Unité de Catalyse et Chimie du Solide, Université de Lille, 59655 Lille, France; 2 Laboratory of Analytical Chemistry, Department of Chemistry, Aristotle University of Thessaloniki, 54124 Thessaloniki, Greece

**Keywords:** deep eutectic solvents, extraction, organic compounds, green sample preparation, analytical chemistry

## Abstract

The use of deep eutectic solvents (DES) is on the rise worldwide because of the astounding properties they offer, such as simplicity of synthesis and utilization, low-cost, and environmental friendliness, which can, without a doubt, replace conventional solvents used in heaps. In this review, the focus will be on the usage of DES in extracting a substantial variety of organic compounds from different sample matrices, which not only exhibit great results but surpass the analytical performance of conventional solvents. Moreover, the properties of the most commonly used DES will be summarized.

## 1. Introduction

Over the last decades, conventional solvents and extraction techniques—for example, methanol as a solvent or liquid–liquid extraction as a technique—tend to be replaced by new, simpler, inexpensive, and environmentally friendly approaches. The movement of “Green Analytical Chemistry” (GAC) and its 12 principles, presented by P. Anastas in 1998, has especially inspired scientists to switch to greener and alternative solutions for extracting compounds from different matrices [1]. Thus, developing new and sustainable solvents to match the above criteria is of high interest to many researchers globally [2,3,4,5,6,7,8]. A new group of organic salts that possess melting points below 100 °C, called ionic liquids (ILs), have emerged and demonstrate properties that could replace volatile organic solvents. However, ionic liquids pose some problems, such as toxicity, stability, biodegradability, and expense regarding their synthesis.

That is where deep eutectic solvents (DES) emerged in 2001 as the perfect candidate for replacing ILs, demonstrating cheaper and easier synthesis, while their environmentally friendliness is much more evident [9,10,11,12]. Deep eutectic solvents have low melting points due to low lattice energy formed by their large and asymmetrical ions. Habitually, they are formed by the combination of metal salt, called the hydrogen bond donor (HBD), with a quaternary ammonium salt, the hydrogen bond acceptor (HBA). Thus, hydrogen bonds are formed by charge delocalization, and the mixture’s melting point is lower than the separate components of the DES [13,14]. Hydrophilic and hydrophobic DES and natural deep eutectic solvents (NADES) can, without a doubt, be applied to analytical chemistry in different fields to extract molecules from a variety of samples [4,8,15,16,17,18,19,20,21,22,23,24,25,26,27,28,29].

**Figure 1:**
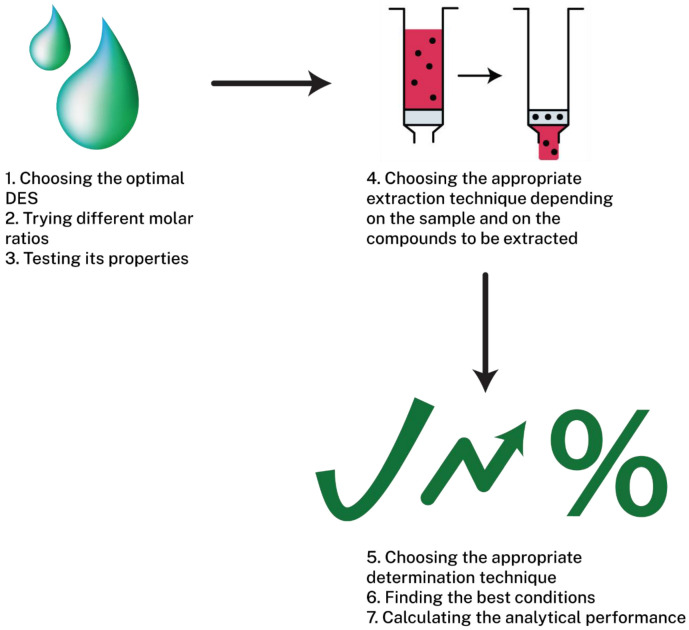
Representative scheme of the typical development of an extraction method by using deep eutectic solvents.

Older reviews on deep eutectic solvents focus, mainly, on the extraction or microextraction techniques [2,4], highlighting their low cost, simplicity, and environmental safeness, on their synthesis and properties [17,20,27,28], or on the variety of samples that they can be applied to [8], such as gas, biodiesel, or metals. The aim of this review is the introduction of deep eutectic solvents and natural deep eutectic solvents and understanding how can they be applied in the extraction of a plethora of compounds, focusing mainly on the different group of organic compounds that can be extracted. Additionally, the synthesis and the properties of these solvents will be discussed briefly. In Figure 1, the main scheme of how DES are used in extraction is presented.

## 2. Synthesis and Properties of Deep Eutectic Solvents

### 2.1. Synthesis of Deep Eutectic Solvents

**Figure 2:**
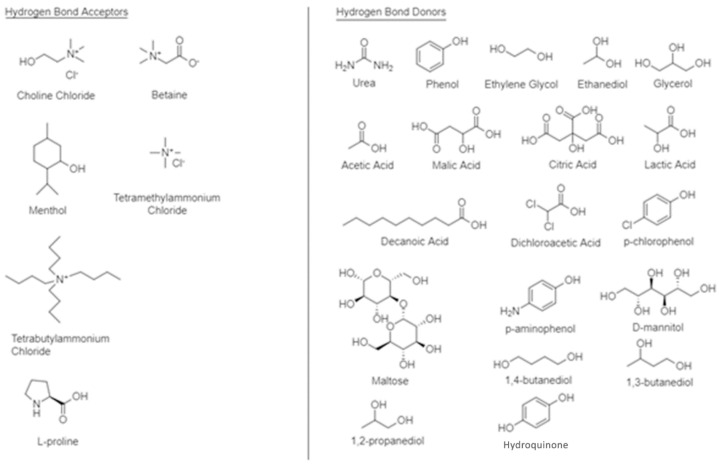
Structures of some common HBAs and hydrogen bond donors HBDs used in the synthesis of deep eutectic solvents.

The most common way to synthesize DES is by mixing two or three cheap and safe salts and heating them up in order to obtain a homogenous solution. Molten salts at the ambient temperature can also be prepared through the mixture of metal salts with quaternary ammonium salts [30,31,32]. There are four main types of deep eutectic solvents: type I, where a metal chloride is combined with a quaternary salt, type II, where a hydrated metal chloride is merged with a quaternary salt, type III, where an HBD is put together with an HBA quaternary salt, and type IV, where an organic HBD compound reacts with a metal chloride [2,33]. Table 1 summarizes the four types of the DES, while in Figure 2, the most frequently used HBA and HBD are presented.

Choline chloride (ChCl) is the most widely used HBA due to its low cost, low toxicity and biodegradability than has the advantage of reacting with safe and inexpensive HBDs, such as carboxylic acids, urea, or glycerol [32,34]. Additionally, type III acidic deep eutectic solvents are synthesized from quaternary organic salts with Brønsted acids (for example, citric acid) and type IV acidic DES from Lewis acids (for instance, zinc chloride or bromide) [35].

### 2.2. Properties of Deep Eutectic Solvents

Physicochemical properties of deep eutectic solvents derive from the various interactions between an HBA and HBD, which depend on the molar ratios, the organic compounds themselves, and the nature of the interactions (π-π and/or hydrogen bonding, anion exchange, weak non-covalent interactions) [36]. The properties that need to be considered when using DES and/or NADES are density, acidity, conductivity, viscosity, volatility, the melting and freezing point, and surface tension. Additionally, the toxicity, cost, thermal stability, and biodegradability should not be passed over [2,37]. The value of pH that results from the combination of HBA and HBD can drastically affect the extraction efficiency of the target compounds, so this parameter ought to not be overlooked as well [38]. As described by El Achkar et al. [39] in a thorough review about the properties of DES, viscosity is an important parameter to be studied, among others, since it severely impacts the extraction efficiency of a developed method. Water content is an ambiguous parameter since, for some, it might seem as an impurity, while others rely on it and add water on purpose to expose the reliability of the DES. Molar ratio and temperature of extraction can impact the isolation of organic compounds as well, and they are studied in almost all developed methods of extraction. Consequently, by knowing the parameters that can affect the extraction with the usage of DES, one can exploit their high tunability [40].

## 3. Extraction of Organic Compounds

### 3.1. Pesticides, Fungicides and Herbicides

Choline chloride (ChCl) with phenol, ethylene glycol, or 4-chlorophenol were used as deep eutectic solvents, in different molar ratios, from Abolghasemi et al. [41] so as to extract triazole fungicides from vegetable samples and fruit juice. The team used headspace single-drop microextraction (HS-SDME) as an extraction technique and gas chromatography coupled with a flame ionization detector (GC-FID) to determine the compounds. By examining a lot of parameters and choosing the optimal ones, they were able to achieve a limit of detections (LODs) of 0.82–1.0 μg/mL and relative standard deviations (RSD) of 3.9–6.2% when ChCl and 4-chlorophenol (1:2, respectively) were used as the DES media.

Farajzadeh et al. [42] synthesized a new DES of high density—consisting of menthol and dichloroacetic acid (1:2 molar ratio)—that was utilized as the extraction solvent of different pesticides originating from honey samples. Dispersive liquid–liquid microextraction (DLLME) was chosen as the technique, while the determination was done by GC-FID. By using these techniques and the aforementioned DES under the best conditions, they were able to detect and quantify the pesticides in the range of 0.32–1.2 ng/g and 1.1–4.0 ng/g, respectively, with enrichment factors (EF) 279–428 and absolute recoveries obtained from the extraction being between 56 and 86%. In another paper, Farajzadeh et al. [43] successfully extracted pesticides from vegetable and fruit juice samples by liquid phase microextraction (LPME) with the help of ChCl and *p*-chlorophenol as the DES. Interestingly, they used different temperatures to disperse the DES into the aqueous phase, thus improving the extraction. EFs were once again very high, in the range of 280–465, with absolute recoveries of 56–93%, and very low LODs and LOQs were achieved (0.13–0.31 and 0.45–1.1 ng/mL respectively).

Pesticides can also be found in wastewater, which poses health risks to the public. According to Florindo et al. [44], a variety of hydrophobic DES were picked to evaluate their removal efficiency of four neonicotinoid pesticides from aqueous environments. The highest extraction efficiency (up to 80%) was accomplished with DL-menthol and organic acids as DES. Another possible source of exposure to pesticides is milk, so the team of Jouyban et al. [45] developed a method to determine some of them by forming the DES in matrix and using them in the liquid phase extraction coupled with the DLLME of solidified organic droplets. ChCl and decanoic acid (molar ratio 1:2) were the best candidates for this extraction, providing LODs of 0.9–3.9 ng/mL, LOQs of 4.8–6.9 ng/mL, EFs of 320–445, and recoveries of 64–89%.

Musarurwa and Tavengwa [46] highlighted the importance of using DES in the liquid–liquid micro-extraction (LLME) so as to successfully extract pesticides from different food samples. One work of Nemati et al. [47] focused on the usage of solidified droplets in DLLME, coupled with stir bar sorptive extraction (SBSE) with the help of water-miscible DES, to extract acidic pesticides from tomato samples. Very high enrichment factors were introduced by this method (2530–2999) and pretty low LODs and LOQs were achieved (7–14 ng/L and 23–47 ng/L, respectively). In another work, Nemati et al. [48] effectively extracted pesticides from milk samples by using tetrabutylammonium chloride (TBAC) and dichloroacetic acid (1:1) as the elution solvents in their DLLME method that they developed based on organic polymers. Once more, low LODs (0.09–0.27 ng/mL) and LOQs (0.31–0.93 ng/mL) were obtained, with EFs of 162–188 and extraction efficiencies of 81–94%.

L-menthol with decanoic acid were used as hydrophobic DES by Lin et al. [49] so as to extract five fungicides in tea and fruit juices by freezing and, therefore, solidifying the DES to use them in ultrasound-assisted DLLME (UA-DLLME). After a thorough examination of the parameters involved in the efficiency of the extraction and, then, using the optimum ones, the relative recoveries were 72–109%, RSDs were 13.5–14.8%, and LODs were 0.75–8.45 μg/mL. Tea samples caught the eye of Torbati et al. [50], who decided to use water-miscible DES to extract and preconcentrate herbicides by LLE that were coupled with in-syringe DLLME in a narrow tube. This method allowed them to accomplish LODs of 2.6–8.4 ng/kg, with EF in the range of 350–445 and absolute recoveries of 70–89%.

### 3.2. Flavonoids, Phenolic and Other Bioactive Compounds

Skarpalezos and Detsi [51] emphasized the significance of DES when selected as the extraction means for flavonoids from different natural sources, including isoflavones, flavones, anthocyanins, flavonois, flavonones, and flavan-3-ols. Choi et al. [52] showed how important it is to use greener approaches to the usage of solvents for the extraction of bioactive compounds from a plethora of natural products, suggesting ILs and DES as the perfect candidates for this task. Plants house a range of bioactive compounds that can be isolated with innovative extraction techniques that involve both DES and natural deep eutectic solvents (NADES), according to Ivanović et al. [53]. DES and NADES can also be used to extract bioactive compounds from agricultural by-products as well, as Socas- Rodríguez et al. [54] highlighted in a recent review. Moreover, a comparison of conventional organic solvents with DES was thoroughly documented by Nakhle et al. [55] regarding, once again, the microextraction of bioactive compounds.

Bi et al. [56] were able to extract two flavonoids, amentoflavone and myricetin, by using ChCl with 1,4-butanediol (1:5 molar ratio) in water and heating at 70 °C for 40 min to get the optimal results. The LODs that were achieved were 0.07 and 0.09 μg/mL, respectively, for the two compounds, and RSDs were 2.72 and 3.06. Nam et al. [57] chose *Flos sophorae*, a common and traditional Chinese medicine, in order to extract flavonoids that are present in this flower, such as kaempferol, quercetin, and isorhamnertin glycosides. By synthesizing a DES of glycerol and L-proline (2:5), by freeze-drying and by applying ultrasound-assisted extraction (UAE), they were able to extract these organic compounds. Other experiments with common solvents, such as methanol, and other extraction techniques—for example, SPE with a C18 cartridge—were evaluated and compared to the usage of DES. It was found that the ultrasound-assisted extraction with DES gave lower absolute recoveries than the SPE method. Nevertheless, the advantages of safety, low-cost, and environmental friendliness that DES provide surpassed the lower extraction efficiency.

Genistein, daidzin, daidzein and genistin, four isoflavones, were successfully extracted with the application of NADES, according to Bajkacz et al. [58]. It was the first time that a solution consisting of 30% ChCl and citric acid with 1:1 molar ratio was used in the ultrasound-assisted extraction of isoflavones from soy products. The determination of the efficiency of the extraction was done by ultra-high performance liquid chromatography coupled with an ultraviolet detector (UHPLC-UV). Enrichment factors reached up to 598, with recoveries between 64.7 and 99.2%. LODs ranged from 0.06 to 0.14 μg/g and RSDs ranged from 1.0 to 5.9%. Meng et al. [59] used the same extraction technique—UAE, with NADES consisting of ChCl:1,2-propanediol (1:4 molar ratio)—so as to extract flavonoids from Pollen Typhae. Compared to methanol and 75% aqueous ethanol, NADES exhibited higher recoveries of 86.87–98.89% for kaempferol, quercetin, isohamnetin, and naringenin, rendering NADES a useful tool for this type of extractions.

Ternary DES, denoting deep-eutectic solvents consisting of the combination of three solvents, were used by Tang et al. [60] to extract two flavonoids from *Ginkgo biloba*. ChCl with oxalic acid and ethylene glycol, at 1:1:3 molar ratio, gave the best extraction efficiencies for myricetin and quercetin under the optimal conditions, which consisted of heating to 60 °C for 30 min. The extracted amount from this plant was 1.11 and 1.40 mg/g of sample for each compound respectively. Flowers of *Abelmoschus Manihot* (*Linn.*) *Medicus* were picked from Wan et al. [61] as the source of bioactive flavonoids that could be extracted with the aid of ChCl and acetic acid (1:2 molar ratio). The target compounds of interest were myricetin, isoquercitrin, and hyperoside, and they were successfully extracted in mild conditions (30 °C, 30 min, 35:1 mg/mL solid-to-solvent ratio), providing 1.11, 5.64, and 11.57 mg of each compound, respectively, per gram of the flower. Quercetin, isohamnetin, and kaempferol were extracted from vegetables with the benefit of betaine-D-mannitol as the ideal DES by Dai and Row [62].

Five Chinese herbal medicinal plants, *Notoginseng Radix* et *Rhizoma*, *Salviae Miltiorrhizae Radix* et *Rhizoma*, *Epimedii Folium*, *Rhei Rhizoma* et *Radix*, and *Berberidis Radix* were selected from the team of Duan et al. [63] to extract flavonoids, anthraquinones, alkaloids, saponins, and phenolic acids as bioactive compounds by applying UAE with DES. A variety of combinations between ChCl, L-proline, and betaine as HBA were examined and tailored for the needs of each extraction. NADES based on ChCl and malic acid caught the eye of Radošević et al. [64], who extracted a variety of bioactive organic compounds from plants, such as anthocyanins and phenolic compounds. Their cytotoxicity was also evaluated in vitro toward two human tumor cell lines, MCF-7 and HeLa. Bioactive compounds, mainly scoparone, quercetin, and rutin, were successfully extracted from Herba *Artemisiae Scopariae* with ILs and DES from Ma et al. [65]. Amounts of 554.32, 899.73, and 10,275.92 µg/g of each compound were able to be extracted under the ideal conditions.

Anthocyanins contained in blueberries were extracted with ternary NADES by da Silva et al. [66]. ChCl:glycerol:citric acid, in molar ratios of 0.5:2:0.5, was used as the NADES that provided similar or higher extraction efficiencies from other conventional organic solvents. Wine lees are another known source of anthocyanins, which Bosiljkov et al. [67] were able to extract by UAE in almost 30 min with the help of the environmentally friendly ChCl-malic acid deep-eutectic solvent. Lactic acid-glucose and ChCl-1,2-propanediol are perfect candidates for the green extraction of anthocyanins from *Catharanthus roseus*, as indicated by Dai et al. [68].

Phenolic compounds are of high importance and interest from scientists all around the world, as they are great antioxidants. Ruesgas-Ramón et al. [69] pinpointed the utilization of DES in the extraction of that group of organic compounds. Redha [70] motivates researchers and suggests to use DES when extracting phenolic compounds from natural sources, as these solvents take into account the principles of Green Analytical Chemistry (GAC). In another study, Dai et al. [71] were able to isolate phenolic metabolites of different polarities from *Carthamus tinctorius* by applying natural deep-eutectic solvents, highly suggesting their usage in the extraction of bioactive compounds from natural sources. Bubalo et al. [72] applied both microwave-assisted and ultrasound-assisted extraction (MAE, UAE) with ChCl-oxalic acid as the DES that was able to extract phenolics from samples of grape skin. Petunidin-3-O-glucoside, delphinidin-3-O-glucoside, cyanidin-3-O-glucoside, malvidin-3-O-glucoside, peonidin-3-O-glucoside, quercetin-3-O-glucoside, and (+)-catechin were the target compounds that were able to be extracted, with LODs ranging from 0.05–0.37 mg/L and impressively low RSD of 0.30–0.96%. García et al. [73] were drawn by the DES as well, especially ChCl-xylitol and ChCl-1,2-propanediol, which they used to extract apigenin, oleacin, oleocanthal, tyrosol, hydroxytyrosol, luteolin, and 1-acetoxypinoresinol from virgin olive oil, increasing the yield to 20–33% and 67.3–68.3%, respectively, for each DES mentioned when compared to conventional organic solvents. Chanioti et al. [74] used NADES for the isolation of phenolic compounds from olive pomace with the usage of non-ordinary extraction techniques, such as microwave-assisted extraction (MAE), UAE, homogenate extraction (HAE), or high hydrostatic pressure-assisted extraction (HHPAE). The NADES that were used for this work were combinations of ChCl with maltose, glycerol, lactic acid, and citric acid. ChCl with lactic acid and citric acid exhibited the best results. Shishov et al. [75] developed a method that involved rotating disk sorptive extraction (RDSE) to extract phenolic compounds from vegetable oil by coating the surface of the disk with ChCl. The DES were formed by the reaction of the coating and the target organic compounds, and then, the phenolic compounds, vanillic acid, tyrosol, gallic acid, thymol, p-coumarinic acid, syringaldehyde, and protocatechuic acid, were eluted in an aqueous phase by the decomposition of the DES. The proposed method exhibited LODs of 10–60 μg/L and absolute recoveries between 66 and 87%.

Alam et al. [76] presented the studies of many scientists around the usage of ChCl-based DES as a possible, greener, and cheaper means of extraction of phenolic compounds from biomass. Orange peel waste was a source of polyphenols that caught the eye of Ozturk et al. [77], who tried and succeeded in extracting them with ChCl-ethylene glycol (1:4 molar ratio). Surprising enough, the peels are high in content of ferulic acid, gallic acid, and p-coumaric acid, which were extracted via solid–liquid extraction (SLE). By-products from agro-foods also contain phenolic compounds that can be isolated by UAE and lactic acid-glucose-water as the DES, according to Fernández et al. [78]. The extraction efficiency was validated by HPLC-DAD, where 14 phenols were evaluated from pear, tomato, and olive by-products. LODs varied from 0.0006 to 0.0891 µg/g, with the time of each analysis taking up to 18 min. El Kantar et al. [79] extracted polyphenols, with lactic acid-glucose as DES, from grapefruit peels by a solid–liquid extraction (SLE) with a 10:1 ratio of liquid to solid. Fu et al. [80] applied pulse-ultrasonication-assisted extraction (P-UAE) with NADES (ChCl-malic acid) as a greener and more efficient approach in order to extract significant phenolic compounds from peels of *Carya cathayensis* Sarg. Wheat waste biomass was chosen by Cherif et al. [81] as the main source of phenolic compounds to be extracted by UAE and, further, thermal treatment of glycerol-citric acid-glycine as the deep-eutectic solvent. With this combination, they managed to isolate 94.62 mg of ferulic acid equivalents per gram of dry mass. Rutin, a flavonoid, could be solubilized more in NADES, based on ChCl or glycerol, than in water—almost 660–1577 times more—and thus, it could be extracted by tartary buckwheat hull with UAE, as instructed by Huang et al. [82]. Additionally, 9.5 mg of rutin per g of sample were able to be isolated, with the extraction efficiency reaching 95%.

In a review, Bubalo et al. [83] introduced the usage of greener solvents for the extraction of biologically active compounds from plants, of which NADES played an important role. Native Greek medicinal plants, mainly sage, mint, dittany, marjoram, and fennel, were selected as the resource of polyphenols to be extracted by Bakirtzi et al. [84] by novel NADES, synthesized from lactic acid with ChCl, ammonium acetate, sodium acetate, or glycine-water in 3:1, 3:1, 3:1 and 3:1:3 molar ratios, respectively. *Lonicerae japonicae flos*, a common Chinese medicinal plant, was the main sample from which five phenolic acids were extracted (caffeic acid, chlorogenic acid, 4,5-dicaffeoylquinic acid, 3,5-dicaffeoylquinic acid, and 3,4-dicaffeoylquinic acid) by Peng et al. [85]. MAE was the main technique used for this feat, while, as DES, ChCl with 1,3-butanediol was used in 1:6 molar ratio. Recoveries for this work ranged from 79.25 to 86.01% for the five organic compounds of interest. Caffeine, rutin, ferulic acid, naringin, rosmarinic acid, and 7-methylrosmanol were extracted from *Rosmarinus officinalis* L. by ChCl-1,2-propanediol in water, providing a total amount of phenolics of 20,588 μg/g. MAE with ChCl-glycerol (1:2) and 20% water constituted the main extraction means for Gao et al. [86] when they developed the method of isolation of phenolic compounds from the leaves of mulberry (*Morus Alba* L.). A total amount of 8.352 mg of phenolics per gram of leaves was able to be extracted, under 18 min at 66 °C, by using 20 mL of DES per gram of sample. Lastly, they utilized a macroporous resin, which was put in a chromatography column to separate the organic compounds of interest from the DES. Park et al. [87] were able to extract two phenolic compounds, caffeic acid and chlorogenic acid, from Herba *Artemisiae Scopariae* by UAE of tetramethyl ammonium chloride-urea (1:4 molar ratio) as DES, which was further mixed with methanol-water (60:40 *v*/*v*). As a liquid-to-solid ratio 10:1 was chosen as the optimal one, which exhibited recoveries of 97.3–100.4%, with RSDs lower than 5%. Furthermore, they were able to extract 0.31 mg and 9.35 mg of each compound, respectively, per gram of sample. Last but not least, Liu et al. [88] used betaine-ethanediol (1:4 molar ration) in 30% water, at 60 °C for 30 min, to extract hesperidin, neohesperidin, naringin, and nariturin from *Aurantii Fructus*. Compared to the methanol extracts, the DES extracts contained higher amounts of isolated organic compounds, namely 3.03, 35.94, 83.98, and 8.39 mg/g. respectively. Some of the organic compounds found in the *Ixora javanica* flower have skin-lightening cosmetic and antioxidant properties, and Oktaviyanti et al. [89] developed a method to extract them. ChCl-propylene glycol, with a 1:1 molar ratio, was the ideal candidate for the application in the UAE of these compounds. The extraction time was only 5 min at 57 °C, and 33 mg of quercetin equivalent per gram of dried sample were able to be obtained.

Pontes et al. [90] used ChCl-acetic acid (1:2 molar ratio) as their ideal DES in the extraction of phenolics from olive leaves, and it was able to extract more phenolic compounds than ethanol under the same conditions. Mogaddam et al. [91] were able to develop a method of extracting three phenolic compounds with organic-free solvents based on DES (tetrabutylammonium chloride-hydroquinone) and by exploiting the LLE/DLLME with heating. LODs reported were 0.13–0.42 ng/mL, EFs were 370–445, and extraction recoveries were from 74 to 89%, with RSDs being equal to or lower than 7.4%. Li et al. [92] tried to tackle the problem of phenols being contained in wastewater, which later result in aqueous environments, by developing a method of isolating them with L-proline-decanoic acid during a 60 min period at 50 °C. The reusage of the NADES that was used was studied, and it was found that it could be reused up to six times, with extraction efficiencies being 62% for the first cycle and 57% up to the sixth cycle.

In Table 2, the methods developed for the extraction of the organic compounds from the first two subgroups are summarized and presented.

### 3.3. Pharmaceutical Compounds and Preservatives

An antibacterial sulfonamide named sulfamerazine was the compound of interest to be extracted by Liu et al. [93], who applied pipette-tip solid-phase extraction (PT-SPE) with graphene modified by DES (ChCl-ethylene glycol) as the adsorbent. The limit of detections that was calculated from this method were 0.01 μg/mL, with relative recoveries of 91.01–96.82% and RSDs below 3.84%. Paracetamol, a common analgetic and antipyretic drug, was able to be isolated from synthetic urea with an inexpensive, new, and simple method by Dogan et al. [94]. A shaker-assisted deep eutectic solvent microextraction (SA-DES-ME) was used as the extraction technique, with betaine-oxalic acid (1:2 molar ratio) playing the role of the DES. The analytical performance of the proposed method was great, with recoveries varying from 94.2–107.1%, LOD of 14.9 µg/L, and very low RSDs (below 3.3%). Li et al. [95] wanted to isolate thiamphenicol and chloromycetin, two antibiotics, from milk in order to purify it. This was achieved by using molecular imprinted polymers (MIPs) in the solid phase extraction (SPE) of the two organic compounds, along with deep eutectic solvents, with 87.02% and 91.23% being the recoveries of the two antibiotics, respectively.

Antiviral organic compounds that are used as drugs to combat viral-related diseases can also be extracted with the use of DES. Jouyban et al. [96] managed to isolate sofosbuvir and daclatasvir from urine with homogenous LLE by applying the DES, p-aminophenol-tetrabutyl ammonium chloride, as a more environmentally friendly alternative that is as effective as conventional solvents. RSDs were lower than 9.3%, low LODs were achieved with sofosbuvir at 1.3 and daclatasvir at 1.0 μg/L, as well as EFs of 90 and 96, respectively, and lastly, extraction recoveries of 90 and 96%. Quinine was successfully able to be extracted via its hydrogen bonding with hydrophilic alcohol-based NADES by Fan et al. [97]. The same NADES could be reused by performing a back-extraction with 1% *v/v* aqueous acetic acid. Acetic acid with menthol served as the deep eutectic solvent to isolate diclofenac from aqueous media. The method developed by Kurtulbaş et al. [98] could reach up to 80% removal of the pharmaceutical compound. Antibiotic residues from honey were extracted with a method developed by Shahi et al. [99] that involved the synthesis of nanocomposites of multiwall carbon nanotubes with urea-formaldehyde and their application in SPE. DES with DLLME were also combined with the above method, aiding in the extraction and the obtaining of improved results, such as LODs of 0.32–0.86 ng/g and LOQs of 1.1–2.9 ng/g, respectively, with the RSD calculated as equal to or lower than 9.1%.

Hadi et al. [100] developed a method to extract tocotrienols and tocopherols from palm oil with the usage of ChCl, with carboxylic acids as the means of extraction. A total of 18,525 mg of tocopherols per kg of sample were able to be isolated with this method. Choline chloride with sucrose, ethylene glycol, and ethanolamine were used in the LLE of α-tocopherols by the team of Mohammadi et al. [101]. α-, γ-, and δ-tocopherols were able to be extracted from soybean oil deodorizer distillate by Liu et al. [102] with phenolic DES, ChCl with p-cresol. The total extraction efficiency of all tocopherols reached 77.6%. Ge et al. [103] managed to synthesize, in situ, the hydrophobic deep eutectic solvent comprised of decanoic acid and DL-menthol, and they used it in the liquid–liquid microextraction (LLME) of four parabens in water samples. With this method, LODs of 0.6–0.8 ng/mL and RSDs less than 7.2% were able to be achieved.

### 3.4. Polycyclic Aromatic Hydrocarbons, Volatile Organic Compounds and Pollutants

Hashemie et al. [104] wrote an extensive review on the usage of ionic liquids and DES in sorptive-based extraction techniques in order to extract environmental pollutants, urging scientists to switch to more greener alternatives for this type of sample preparation. Among these pollutants, PAHs play an important role.

In one interesting work, Shakirova et al. [105] used fatty acids that came from the in situ alkaline hydrolysis of triglycerides in milk so as to synthesize NADES between them, with menthol or thymol being the second component. With this method, they were able to extract thirteen polycyclic aromatic compounds (PAHs) from milk: pyrene, anthracene, phenanthrene, chrysene, fluoranthene, fluorene, naphthalene, benz[*α*]anthracene, benzo[*α*]pyrene, benzo[*b*]fluoranthene, benzo[*k*]fluoranthene, benzo[*ghi*]perylene, and dibenz[*α,h*]anthracene. LODs varied from 0.002–0.09 μg/kg, with 70–91% being the extraction efficiencies and RSDs being lower than 5.2%. Nie et al. [106] chose volatile organic compounds (VOCs) as their compounds of interest to be extracted from tobacco, utilizing MAE with a DES that was coupled with headspace solid phase microextraction (HS-SPME), followed by gas chromatography with a mass spectrometer (GC-MS) for their determination.

### 3.5. Polysaccharides, Pigments and Terpenes

A famous Chinese plant that has many invigorating health benefits when consumed, *Dioscorea opposita* Thunb, caught the eye of Zhang and Wang [107] who developed a method of extraction of polysaccharides by using UAE with ChCl and 1,4-butanediol, constituting the deep eutectic solvent. Under the best conditions, with the temperature reaching 94 °C, the water content of the extraction with the DES being 32.89% *v/v*, and the extraction time being less than 45 min, they were able to get the best results. Shang et al. [108] used MAE with the same DES, ChCl-1,4-butanediol with 1:5 molar ratio, in order to be able to extract polysaccharides from *Fucus vesiculosus*, attaining 116.33 mg of the target compounds per gram of sample. Optimal conditions for this isolation were 35 min of extraction at 168 °C, with 32% content of water. Furthermore, the in vitro evaluation assay of their biological activity was done in HeLa cells, highlighting their antioxidant activity. Edible brown seaweed, called *Sargassum horneri*, is another source of polysaccharides that attracted Nie et al. [109]. With their method that consisted of the usage of ChCl-1,2-propanediol with 1:2 molar ratio in 30% of water (*v/v*), of the heating to 70 °C, and of the solid-to-liquid ratio reaching 1:30 g/mL, they managed to extract them with high efficiencies. Once again, the in vitro biological activity was assessed. Das et al. [110] developed a method of isolating κ-carrageenan from *Kappaphycus alvarezii* that involved hydrated DES, with 10% hydrated ChCl-glycerol (1:2 molar ratio) being the best candidate for the extraction, giving a yield of 60.25%.

Citric acid with glucose, with 1:1 molar ratio, served as the natural deep eutectic solvent for Liu et al. [111] who wanted to extract natural pigments, such as curcumin, demethoxycurcumin, and bis-demethoxycurcumin, from *Curcuma longa* L. Subsequently, they performed SPE to recover the NADES and reuse it. This method exhibited impressively low RSDs, 0.46–0.91%, and LODs ranging from 0.25 to 0.37 mg/L. Patil et al. [112] proposed a green approach of extracting curcuminoids from *Curcuma longa* by UAE with DES, namely ChCl-lactic acid, with a 1:1 molar ratio. The maximum amount of curcuminoids that were able to be extracted was 77.13 mg/g. Curcumin was extracted and preconcentrated from herbal tea and food samples by a LLME emulsification of DES, and its performance was determined by UV-vis, as reported by Aydin et al. [113]. The pH that was selected for this method was the value 4, while other parameters were also studied. Under the best conditions, LODs were 2.86 µg/L, RSDs were lower than 6%, and recoveries varied from 96 to 102%. Zhang et al. [114] extracted astaxanthin from shrimp by-products by applying a UAE with DES and compared this method with the extraction using ethanol. It was found that the proposed method was able to obtain more of the organic compound than with the conventional solvent (146 μg/g with DES and 102 μg/g with ethanol). Rhodamine B, a colorant that is no longer used, by law, due to its carcinogenic properties, was isolated from chili oil by Wang et al. [115] by doing an extraction with ChCl-ethylene glycol and determining the efficiency by ultra-high performance liquid chromatography coupled with a fluorescence detector (UHPLC-FD).

Su et al. [116] applied UAE with two different DES, ChCl-urea and betaine-ethylene glycol, to extract terpene trilactones from the leaves of *Ginkgo biloba*. The latter DES worked best and gave the highest extraction efficiencies that reached 99.37% and 1.94 mg/g.

### 3.6. Other Organic Compounds

Benzophenone-type UV filters can be extracted and preconcentrated with the benefit of hydrophobic DES by an air-assisted dispersive liquid–liquid mixroextraction (AA-DLLME) from aqueous samples, as reported by Ge et al. [117]. DL-menthol with decanoic acid was used as the deep eutectic solvent and the proposed method was applied successfully, providing 88.8–105.9% relative recoveries. There were two common organic solvents, acetonitrile and butanol, extracted from aqueous solutions, with LLE and DES by Rabhi et al. [118], by forming ternary systems.

An environmentally friendly extraction method that involved ChCl-1,3-butanediol in the ball mill-assisted extraction of tanshinones (tanshinone I, tanshinone II A, and cryptotanshinone) from plants was developed by Wang et al. [119]. Extracted amounts reached 0.181, 0.421, and 0.176 mg/g respectively for each compound, with RSDs lower than 1.9%, recoveries of 96.1–103.9%, and LODs of 5–8 ng/mL being reported. Catechin compounds, such as (+)-epicatechin gallate, (−)-epigallocatechin gallate, and catechin were extracted from Chinese green tea by the usage of deep eutectic solvents. Zhang et al. [120], with this method, demonstrated an eco-friendly pathway that could achieve 35.25, 114.2, and 3.629 mg of an extracted compound, respectively, per gram of sample, with extraction efficiencies ranging from 82.7 to 97.0%.

Zhao et al. [121] extracted the essential oil out of cumin seeds (*Cuminum cyminum* L.) by a MAE that involved NADES and was done in three steps. ChCl-L-lactic acid (1:3 molar ratio) was the ideal candidate for this work, which helped this team to extract 58 certified essential oil components, with the major components consisting of cuminol, moslene, terpineol, and cuminal. The highest yield reported was 2.22% of essential oil from the seeds. MAE, with NADES consisting of ChCl and oxalic acid (1:1 molar ratio), were involved in the extraction of essential oil from turmeric (*Curcuma longa* L.). The yield that was obtained was 0.85%, while 49 organic compounds were determined by GC-MS [122].

Milani et al. [123] chose tetraethylammonium chloride with ethylene glycol, at 1:2 molar ratio, as their deep eutectic solvent in the UAE of rebaudioside A and stevioside, two steviol glycosides found in *Stevia rebaudiana*. Tests with conventional extraction methods showed that the proposed method was three times more efficient.

In Table 3, all the discussed methods from Section 3.3, Section 3.4, Section 3.5 and Section 3.6 are summarized, and their analytical performances are demonstrated.

## 4. Conclusions

As discussed herein, deep eutectic solvents and natural deep eutectic solvents can have a vast variety of applications in the extraction and preconcentration of organic compounds. Some of the groups of organic compounds that were isolated by their usage involve pesticides, fungicides, herbicides, bioactive compounds, flavonoids, phenolic and pharmaceutical compounds, pigments, terpenes, polycyclic aromatic compounds, volatile organic compounds and other pollutants, preservatives, or other organic compounds.

This quite big range of possible extracted compounds renders DES an important tool to be exploited. Additionally, they offer both high absolute and relative recoveries when coupled with extraction or microextraction techniques, very low limits of detection and quantification, as well as quite low relative standard deviations. Additionally, their low cost, environmental friendliness, and simplicity attract researchers to use them and to find new combinations that can combat conventional organic solvents that pose risks to the health of the user or to organisms in general. Their properties are still being studied, and hopefully, their commercialization will soon be done.

## Figures and Tables

**Table 1:** The four types of Deep Eutectic Solvents and their formulas.

Type	Formula	Terms
I	Cat^+^X^−^ + zMCl_x_	M = Zn, Sn, Ga, In, Al, Fe
II	Cat^+^X^−^ + zMCl_x_ · yH_2_O	M = Cr, Cu, Fe, Ni, Co
III	Cat^+^X^−^ + zRZ	Z = COOH, CONH_2_, OH
IV	MCl_x_ + RZ	M = Al, Zn and Z = CONH_2_, OH

Cat^+^ = any phosphonium, ammonium or sulfonium cation, X^−^ = a Lewis base, often a halide anion, MCl_x_ = metal chloride, RZ = organic compound.

**Table 2:** Summary and analytical performance of the methods discussed in Section 3.1 and Section 3.2.

Group of Compounds	DES	Molar Ratio	Extraction Technique	Recovery (%)	LOD ^1^	Reference
Triazole fungicides	ChCl: 4-chlorophenol	1:2	HS-SDME	94–97	0.82–1.0 mg/L	[41]
Pesticides	Menthol: dichloroacetic acid	1:2	DLLME	56–86	0.32–1.2 ng/g	[42]
Pesticides	ChCl: p-chlorophenol	1:2	LPME	56–93	0.13–0.31 ng/mL	[43]
Neonicotinoid pesticides	Menthol: dodecanoic acid	2:1	LLE	80	N/R ^2^	[44]
Pesticides	ChCl: decanoic acid	1:2	DLLME	64–89	0.9–3.9 ng/mL	[45]
Acidic pesticides	ChCl: ethylene glycol	1:2	DLLME-SBSE	76–90	7–14 ng/L	[47]
Pesticides	TBAC: dichloroacetic acid	1:1	DLLME	81–94	0.09–0.27 ng/mL	[48]
Fungicides	Menthol: decanoic acid	1:1	UA-DLLME	72–109	0.75–8.45 μg/mL	[49]
Herbicides	ChCl: butyric acid	1:2	LLE-DLLME	70–89	2.6–8.4 ng/kg	[50]
Flavonoids	ChCl: 1,4-butanediol	1:5	UAE	N/R	0.07, 0.09 μg/mL	[56]
Flavonoids	Glycerol: L-proline	2:5	UAE, SPE	81–87	N/R	[57]
Isoflavones	ChCl: citric acid	1:1	UAE	64.7–99.2	0.06–0.14 μg/mL	[58]
Flavonoids	ChCl: 1,2-propanediol	1:4	UAE	86.7–98.9	0.05–0.14 μg/mL	[59]
Flavonoids	ChCl: oxalic acid: ethylene glycol	1:1:3	LLE	N/R	1.11–1.40 mg/g	[60]
Flavonoids	ChCl: acetic acid	1:2	UAE	N/R	1.11–11.57 mg/g	[61]
Flavonoids	Betaine: D-mannitol	N/R	UAE	91.7–95.8	0.14–0.17 μg/mL	[62]
Flavonoids	Different types	Diff. ^3^	UAE	93.8–107.7	0.14–0.22 μg/mL	[63]
Bioactive compounds	ChCl: malic acid	1:1	UAE	N/R	N/R	[64]
Bioactive compounds	ILs, ChCl based DES	Diff.	Reflux	N/R	N/R	[65]
Anthocyanins	ChCl: glycerol:citric acid	0.5:2:0.5	UAE	N/R	0.02 mg/L	[66]
Anthocyanins	ChCl: malic acid	Diff.	UAE	N/R	0.15–0.28 mg/L	[67]
Anthocyanins	Lactic acid: glucose	1:2	Stirring	N/R	N/R	[68]
Phenolic metabolites	Different types	Diff.	N/R	75–97	N/R	[71]
Phenolics	ChCl: oxalic acid	1:1	MAE, UAE	N/R	0.05–0.37 mg/L	[72]
Phenolics	ChCl: 1,2-propanediol	1:1	Vortex	N/R	N/R	[73]
Phenolics	ChCl: lactic acid	1:2	MAE, HUE, UAE	N/R	N/R	[74]
Phenolics	ChCl + phenolics	N/R	RDSE	66–87	10–60 μg/L	[75]
Polyphenols	ChCl: ethylene glycol	1:4	SLE	N/R	N/R	[77]
Phenolics	Lactic acid: glucose	5:1	UAE	86.0–109.6	0.6–89.1 ng/g	[78]
Polyphenols	Lactic acid: glucose	5:1	SLE	N/R	N/R	[79]
Phenolics	ChCl: malic acid	1.5:1	P-UAE	N/R	N/R	[80]
Phenolics	Glycerol: citric acid: glycine	Diff.	UAE	N/R	N/R	[81]
Flavonoids	ChCl: glycerol	1:1	UAE	95	N/R	[82]
Polyphenols	Lactic acid: glycine:water	3:1:3	UAE	N/R	N/R	[84]
Phenolic acids	ChCl: 1,3-butanediol	1:6	MAE	79.2–86.0	N/R	[85]
Phenolics	ChCl: glycerol	1:2	MAE	77.8–83.8	0.15–0.78 μg/mL	[86]
Phenolics	Tetramethyl ammonuium chloride:urea	1:4	UAE	97.3–100.4	N/R	[87]
Flavanones	Betaine: ethanediol	1:4	Heated LLE	97.0–101.6	N/R	[88]
Bioactive compounds	ChCl: propylene glycol	1:1	UAE	N/R	N/R	[89]
Phenolics	ChCl: acetic acid	1:2	Thermo-shaking	N/R	N/R	[90]
Phenolics	TBAC: hydroquinone	1:2	LLE/DLLME	74–89	0.13–0.42 ng/mL	[91]
Phenols	L-proline: decanoic acid	1:4.2	Stirring	57–62	N/R	[92]

^1^ Limit of Detection, ^2^ Not reported, ^3^ Different molar ratios tested.

**Table 3:** Summary and analytical performance of the methods discussed from Section 3.3, Section 3.4, Section 3.5 and Section 3.6.

Group of Compounds	DES	Molar Ratio	Extraction Technique	Recovery (%)	LOD ^1^	Reference
Sulfonamide	ChCl: ethylene glycol	1:2	PT-SPE	91.0–96.8	0.01 μg/mL	[93]
Analgetic	Betaine: oxalic acid	1:2	SA-DES-ME	94.2–107.1	14.9 μg/L	[94]
Antibiotics	ChCl: glycerol	1:2	MIPs-SPE	87.0–91.2	N/R ^2^	[95]
Antivirals	TBAC: p-aminophenol	1:2	LLE	90–96	1.0–1.3 μg/L	[96]
Antimalarial	Menthol: fenchyl alcohol	1:1	Stirring	101	N/R	[97]
NSAID ^3^	Menthol: acetic acid	Diff. ^4^	LLE	80	N/R	[98]
Antibiotics	TBAC: butanol	1:1	SPE, DLLME	84–99	0.32–0.86 ng/g	[99]
Tocotrienols, tocopherols	ChCl: malonic acid	1:1	LLE	93.0–99.8	N/R	[100]
α-tocopherols	ChCl: sucrose	1:2	LLE	N/R	N/R	[101]
α-, γ-, δ-tocopherols	ChCl: *p*-cresol	1:2	Vortex	77.6	N/R	[102]
Parabens	Menthol: decanoic acid	2:1	LLME	69.1–78.5	0.6–0.8 mg/mL	[103]
PAHs	Menthol or thymol with fatty acids	Diff.	Stirring	70–91	2–90 ng/kg	[105]
VOCs	ChCl: urea	1:3	MAE, HS-SPME	N/R	N/R	[106]
Polysaccharides	ChCl: 1,4-butanediol	N/R	UAE	N/R	N/R	[107]
Polysaccharides	ChCl: 1,4-butanediol	1:5	MAE	91.2	N/R	[108]
Polysaccharides	ChCl: 1,2-propanediol	1:2	UAE	N/R	N/R	[109]
Polysaccharides	ChCl: glycerol	1:2	Thermal treatment	60.3	N/R	[110]
Natural pigments	Citric acid: glucose	1:1	SPE	88.5–94.4	0.25–0.37 mg/L	[111]
Curcuminoids	ChCl: lactic acid	1:1	UAE	N/R	N/R	[112]
Curcuminoids	ChCl: phenol	1:4	VA-LLME	96–102	2.86 μg/L	[113]
Pigment	Different types	Diff.	UAE	N/R	N/R	[114]
Pigment	ChCl: ethylene glycol	Diff.	N/R	N/R	N/R	[115]
Terpene trilactones	Betaine: ethylene glycol	Diff.	UAE	99.4	N/R	[116]
Benzophenone-type UV filters	Menthol: decanoic acid	1:1	AA-DLLME	88.8–105.9	0.05–0.2 ng/mL	[117]
Organic solvents	Menthol: capric acid	2:1	LLE	N/R	N/R	[118]
Tanshinones	ChCl: -1,3-butanediol	N/R	BMAE	96.1–103.9	5–8 ng/mL	[119]
Catechins	Different types	Diff.	LLE	82.7–97.0	N/R	[120]
Essential oils	ChCl: L-lactic acid	1:3	MAE	N/R	N/R	[121]
Essential oils	ChCl: oxalic acid	1:1	MAE	N/R	N/R	[122]
Steviol glycosides	Tetraethylammonium chloride: ethylene glycol	1:2	UAE	N/R	N/R	[123]

^1^ Limit of Detection, ^2^ Not reported, ^3^ Non-steroidal anti-inflammatory drugs, ^4^ Different molar ratios tested.

## Data Availability

Not applicable.

## References

[1] Armenta S., Garrigues S., Esteve-Turrillas F.A., de la Guardia M. (2019). Green extraction techniques in green analytical chemistry. Trends Anal. Chem..

[2] Plastiras O.E., Andreasidou E., Samanidou V. (2020). Microextraction techniques with deep eutectic solvents. Molecules.

[3] Paiva A., Craveiro R., Aroso I., Martins M., Reis R.L., Duarte A.R.C. (2014). Natural deep eutectic solvents—Solvents for the 21st century. ACS Sustain. Chem. Eng..

[4] Cunha S.C., Fernandes J.O. (2018). Extraction techniques with deep eutectic solvents. Trends Anal. Chem..

[5] Espino M., Fernandez M.D., Gomez F.J.V., Silva M.F. (2016). Natural designer solvents for greening analytical chemistry. Trends Anal. Chem..

[6] Vanda H., Dai Y.T., Wilson E.G., Verpoorte R., Choi Y.H. (2018). Green solvents from ionic liquids and deep eutectic solvents to natural deep eutectic solvents. Comptes Rendus Chim..

[7] Florindo C., Branco L.C., Marrucho I.M. (2019). Quest for green-solvent design: From hydrophilic to hydrophobic (Deep) eutectic solvents. ChemSusChem.

[8] Tang B., Zhang H., Row K.H. (2015). Application of deep eutectic solvents in the extraction and separation of target compounds from various samples. J. Sep. Sci..

[9] Kissoudi M., Samanidou V. (2018). Recent advances in applications of ionic liquids in miniaturized microextraction techniques. Molecules.

[10] Pena-Pereira F., Namiesnik J. (2014). Ionic liquids and deep eutectic mixtures: Sustainable solvents for extraction processes. ChemSusChem.

[11] Romero A., Santos A., Tojo J., Rodriguez A. (2008). Toxicity and biodegradability of imidazolium ionic liquids. J. Hazard. Mater..

[12] Berthod A., Ruiz-Angel M.J., Carda-Broch S. (2018). Recent advances on ionic liquid uses in separation techniques. J. Chromatogr. A.

[13] Smith E.L., Abbott A.P., Ryder K.S. (2014). Deep Eutectic Solvents (DESs) and their applications. Chem. Rev..

[14] Abbott A.P., Capper G., Davies D.L., Munro H.L., Rasheed R.K., Tambyrajah V. (2001). Preparation of novel, moisture-stable, Lewis-acidic ionic liquids containing quaternary ammonium salts with functional side chains. Chem. Commun..

[15] Shishov A., Bulatov A., Locatelli M., Carradori S., Andruch V. (2017). Application of deep eutectic solvents in analytical chemistry. A review. Microchem. J..

[16] Dai Y.T., van Spronsen J., Witkamp G.J., Verpoorte R., Choi Y.H. (2013). Natural deep eutectic solvents as new potential media for green technology. Anal. Chim. Acta.

[17] Hansen B.B., Spittle S., Chen B., Poe D., Zhang Y., Klein J.M., Horton A., Adhikari L., Zelovich T., Doherty B.W. (2021). Deep eutectic solvents: A review of fundamentals and applications. Chem. Rev..

[18] Dai Y.T., van Spronsen J., Witkamp G.J., Verpoorte R., Choi Y.H. (2013). Ionic liquids and deep eutectic solvents in natural products research: Mixtures of solids as extraction solvents. J. Nat. Prod..

[19] Tang W., An Y., Row K.H. (2021). Emerging applications of (micro) extraction phase from hydrophilic to hydrophobic deep eutectic solvents: Opportunities and trends. Trends Anal. Chem..

[20] van Osch D., Dietz Chjt van Spronsen J., Kroon M.C., Gallucci F., Annaland M.V., Tuinier R. (2019). A search for natural hydrophobic deep eutectic solvents based on natural components. ACS Sustain. Chem. Eng..

[21] Zdanowicz M., Wilpiszewska K., Spychaj T. (2018). Deep eutectic solvents for polysaccharides processing. A review. Carbohydr. Polym..

[22] Makos P., Slupek E., Gebicki J. (2020). Hydrophobic deep eutectic solvents in microextraction techniques-A review. Microchem. J..

[23] Dwamena A.K. (2019). Recent advances in hydrophobic deep eutectic solvents for extraction. Separations.

[24] Santana-Mayor A., Rodriguez-Ramos R., Herrera-Herrera A.V., Socas-Rodriguez B., Rodriguez-Delgado M.A. (2021). Deep eutectic solvents. The new generation of green solvents in analytical chemistry. Trends Anal. Chem..

[25] Yang Z., Itoh T., Koo Y.M. (2019). Natural Deep Eutectic Solvents and Their Applications in Biotechnology. Application of Ionic Liquids in Biotechnology.

[26] Chen J.N., Li Y., Wang X.P., Liu W. (2019). Application of deep eutectic solvents in food analysis: A review. Molecules.

[27] Zhao R.T., Pei D., Yu P.L., Wei J.T., Wang N.L., Di D.L., Liu Y.W. (2020). Aqueous two-phase systems based on deep eutectic solvents and their application in green separation processes. J. Sep. Sci..

[28] Li L.N., Liu Y.M., Wang Z.T., Yang L., Liu H.W. (2021). Development and applications of deep eutectic solvent derived functional materials in chromatographic separation. J. Sep. Sci..

[29] Tang B.K., Row K.H. (2013). Recent developments in deep eutectic solvents in chemical sciences. Mon. Chem..

[30] Abbott A.P., Boothby D., Capper G., Davies D.L., Rasheed R.K. (2004). Deep eutectic solvents formed between choline chloride and carboxylic acids: Versatile alternatives to ionic liquids. J. Am. Chem. Soc..

[31] Sereshti H., Jamshidi F., Nouri N., Nodeh H.R. (2020). Hyphenated dispersive solid- and liquid-phase microextraction technique based on a hydrophobic deep eutectic solvent: Application for trace analysis of pesticides in fruit juices. J. Sci. Food Agric..

[32] Zhang Q.H., Vigier K.D., Royer S., Jerome F. (2012). Deep eutectic solvents: Syntheses, properties and applications. Chem. Soc. Rev..

[33] Abbott A.P., Capper G., Davies D.L., Rasheed R.K., Tambyrajah V. (2003). Novel solvent properties of choline chloride/urea mixtures. Chem. Commun..

[34] Florindo C., Oliveira F.S., Rebelo L.P.N., Fernandes A.M., Marrucho I.M. (2014). Insights into the synthesis and properties of deep eutectic solvents based on cholinium chloride and carboxylic acids. ACS Sustain. Chem. Eng..

[35] Qin H., Hu X.T., Wang J.W., Cheng H.Y., Chen L.F., Qi Z.W. (2020). Overview of acidic deep eutectic solvents on synthesis, properties and applications. Green Energy Environ..

[36] Chen J., Liu M.J., Wang Q., Du H.Z., Zhang L.W. (2016). Deep Eutectic solvent-based microwave-assisted method for extraction of hydrophilic and hydrophobic components from radix salviae miltiorrhizae. Molecules.

[37] Dai Y.T., Witkamp G.J., Verpoorte R., Choi Y.H. (2015). Tailoring properties of natural deep eutectic solvents with water to facilitate their applications. Food Chem..

[38] Skulcova A., Russ A., Jablonsky M., Sima J. (2018). The pH behavior of seventeen deep eutectic solvents. Bioresources.

[39] El Achkar T., Greige-Gerges H., Fourmentin S. (2021). Basics and properties of deep eutectic solvents: A review. Environ. Chem..

[40] Liu Y., Friesen J.B., McAlpine J.B., Lankin D.C., Chen S.N., Pauli G.F. (2018). Natural deep eutectic solvents: Properties, applications, and perspectives. J. Nat. Prod..

[41] Abolghasemi M.M., Piryaei M., Imani R.M. (2020). Deep eutectic solvents as extraction phase in head-space single-drop microextraction for determination of pesticides in fruit juice and vegetable samples. Microchem. J..

[42] Farajzadeh M.A., Abbaspour M., Kazemian R. (2019). Synthesis of a green high density deep eutectic solvent and its application in microextraction of seven widely used pesticides from honey. J. Chromatogr. A.

[43] Farajzadeh M.A., Hojghan A.S., Mogaddam M.R.A. (2018). Development of a new temperature-controlled liquid phase microextraction using deep eutectic solvent for extraction and preconcentration of diazinon, metalaxyl, bromopropylate, oxadiazon, and fenazaquin pesticides from fruit juice and vegetable samples followed by gas chromatography-flame ionization detection. J. Food Compos. Anal..

[44] Florindo C., Branco L.C., Marrucho I.M. (2017). Development of hydrophobic deep eutectic solvents for extraction of pesticides from aqueous environments. Fluid Phase Equilib..

[45] Jouyban A., Farajzadeh M.A., Mogaddam M.R.A. (2020). In matrix formation of deep eutectic solvent used in liquid phase extraction coupled with solidification of organic droplets dispersive liquid-liquid microextraction; application in determination of some pesticides in milk samples. Talanta.

[46] Musarurwa H., Tavengwa N.T. (2021). Deep eutectic solvent-based dispersive liquid-liquid micro-extraction of pesticides in food samples. Food Chem..

[47] Nemati M., Farajzadeh M.A., Mohebbi A., Khodadadeian F., Mogaddam M.R.A. (2020). Development of a stir bar sorptive extraction method coupled to solidification of floating droplets dispersive liquid-liquid microextraction based on deep eutectic solvents for the extraction of acidic pesticides from tomato samples. J. Sep. Sci..

[48] Nemati M., Tuzen M., Farazajdeh M.A., Kaya S., Mogaddam M.R.A. (2022). Development of dispersive solid-liquid extraction method based on organic polymers followed by deep eutectic solvents elution; application in extraction of some pesticides from milk samples prior to their determination by HPLC-MS/MS. Anal. Chim. Acta.

[49] Lin Z.H., Zhang Y.H., Zhao Q.Y., Chen A.H., Jiao B.N. (2021). Ultrasound-assisted dispersive liquid-phase microextraction by solidifying L-menthol-decanoic acid hydrophobic deep eutectic solvents for detection of five fungicides in fruit juices and tea drinks. J. Sep. Sci..

[50] Torbati M., Farajzadeh M.A., Mogaddam M.R.A. (2019). Deep eutectic solvent based homogeneous liquid-liquid extraction coupled with in-syringe dispersive liquid-liquid microextraction performed in narrow tube; application in extraction and preconcentration of some herbicides from tea. J. Sep. Sci..

[51] Skarpalezos D., Detsi A. (2019). Deep eutectic solvents as extraction media for valuable flavonoids from natural sources. Appl. Sci..

[52] Choi Y.H., Verpoorte R. (2019). Green solvents for the extraction of bioactive compounds from natural products using ionic liquids and deep eutectic solvents. Curr. Opin. Food Sci..

[53] Ivanovic M., Razborsek M.I., Kolar M. (2020). Innovative extraction techniques using deep eutectic solvents and analytical methods for the isolation and characterization of natural bioactive compounds from plant material. Plants.

[54] Socas-Rodriguez B., Torres-Cornejo M.V., Alvarez-Rivera G., Mendiola J.A. (2021). Deep eutectic solvents for the extraction of bioactive compounds from natural sources and agricultural by-products. Appl. Sci..

[55] Nakhle L., Kfoury M., Mallard I., Landy D., Greige-Gerges H. (2021). Microextraction of bioactive compounds using deep eutectic solvents: A review. Environ. Chem. Lett..

[56] Bi W.T., Tian M.L., Row K.H. (2013). Evaluation of alcohol-based deep eutectic solvent in extraction and determination of flavonoids with response surface methodology optimization. J. Chromatogr. A.

[57] Nam M.W., Zhao J., Lee M.S., Jeong J.H., Lee J. (2015). Enhanced extraction of bioactive natural products using tailor-made deep eutectic solvents: Application to flavonoid extraction from Flos sophorae. Green Chem..

[58] Bajkacz S., Adamek J. (2017). Evaluation of new natural deep eutectic solvents for the extraction of isoflavones from soy products. Talanta.

[59] Meng Z.R., Zhao J., Duan H.X., Guan Y.Y., Zhao L.S. (2018). Green and efficient extraction of four bioactive flavonoids from Pollen Typhae by ultrasound-assisted deep eutectic solvents extraction. J. Pharm. Biomed..

[60] Tang W.Y., Li G.Z., Chen B.Q., Zhu T., Row K.H. (2017). Evaluating ternary deep eutectic solvents as novel media for extraction of flavonoids from Ginkgo biloba. Sep. Sci. Technol..

[61] Wan Y.Y., Wang M., Zhang K.L., Fu Q.F., Wang L.J., Gao M.J., Xia Z.N., Gao D.E. (2019). Extraction and determination of bioactive flavonoids from *Abelmoschus manihot* (Linn.) Medicus flowers using deep eutectic solvents coupled with high-performance liquid chromatography. J. Sep. Sci..

[62] Dai Y., Row K.H. (2019). Application of natural deep eutectic solvents in the extraction of quercetin from vegetables. Molecules.

[63] Duan L., Dou L.L., Guo L., Li P., Liu E.H. (2016). Comprehensive evaluation of deep eutectic solvents in extraction of bioactive natural products. ACS Sustain. Chem. Eng..

[64] Radosevic K., Curko N., Srcek V.G., Bubalo M.C., Tomasevic M., Ganic K.K., Redovnikovic I.R. (2016). Natural deep eutectic solvents as beneficial extractants for enhancement of plant extracts bioactivity. LWT Food Sci. Technol..

[65] Ma W., Row K.H. (2017). Optimized extraction of bioactive compounds from Herba Artemisiae Scopariae with ionic liquids and deep eutectic solvents. J. Liq. Chromatogr. Relat. Technol..

[66] da Silva D.T., Pauletto R., Cavalheiro S.D., Bochi V.C., Rodrigues E., Weber J., da Silva C.D., Morisso F.D., Barcia M.T., Emanuelli T. (2020). Natural deep eutectic solvents as a biocompatible tool for the extraction of blueberry anthocyanins. J. Food Compos. Anal..

[67] Bosiljkov T., Dujmic F., Bubalo M.C., Hribar J., Vidrih R., Brncic M., Zlatic E., Redounikavic I.R., Jokic S. (2017). Natural deep eutectic solvents and ultrasound-assisted extraction: Green approaches for extraction of wine lees anthocyanins. Food Bioprod. Process..

[68] Dai Y.T., Rozema E., Verpoorte R., Choi Y.H. (2016). Application of natural deep eutectic solvents to the extraction of anthocyanins from Catharanthus roseus with high extractability and stability replacing conventional organic solvents. J. Chromatogr. A.

[69] Ruesgas-Ramon M., Figueroa-Espinoza M.C., Durand E. (2017). Application of Deep Eutectic Solvents (DES) for phenolic compounds extraction: Overview, challenges, and opportunities. J. Agric. Food Chem..

[70] Redha A.A. (2021). Review on extraction of phenolic compounds from natural sources using green deep eutectic solvents. J. Agric. Food Chem..

[71] Dai Y.T., Witkamp G.J., Verpoorte R., Choi Y.H. (2013). Natural deep eutectic solvents as a new extraction media for phenolic metabolites in *carthamus tinctorius* L.. Anal. Chem..

[72] Bubalo M.C., Curko N., Tomasevic M., Ganic K.K., Redovnikovic I.R. (2016). Green extraction of grape skin phenolics by using deep eutectic solvents. Food Chem..

[73] Garcia A., Rodriguez-Juan E., Rodriguez-Gutierrez G., Rios J.J., Fernandez-Bolanos J. (2016). Extraction of phenolic compounds from virgin olive oil by deep eutectic solvents (DESs). Food Chem..

[74] Chanioti S., Tzia C. (2018). Extraction of phenolic compounds from olive pomace by using natural deep eutectic solvents and innovative extraction techniques. Innov. Food Sci. Emerg. Technol..

[75] Shishov A., Volodina N., Gagarionova S., Shilovskikh V., Bulatov A. (2021). A rotating disk sorptive extraction based on hydrophilic deep eutectic solvent formation. Anal. Chim. Acta.

[76] Alam M.A., Muhammad G., Khan M.N., Mofijur M., Lv Y.K., Xiong W.L., Xu J.L. (2021). Choline chloride-based deep eutectic solvents as green extractants for the isolation of phenolic compounds from biomass. J. Clean. Prod..

[77] Ozturk B., Parkinson C., Gonzalez-Miquel M. (2018). Extraction of polyphenolic antioxidants from orange peel waste using deep eutectic solvents. Sep. Purif. Technol..

[78] Fernandez M.D., Espino M., Gomez F.J.V., Silva M.F. (2018). Novel approaches mediated by tailor-made green solvents for the extraction of phenolic compounds from agro-food industrial by-products. Food Chem..

[79] El Kantar S., Rajha H.N., Boussetta N., Vorobiev E., Maroun R.G., Louka N. (2019). Green extraction of polyphenols from grapefruit peels using high voltage electrical discharges, deep eutectic solvents and aqueous glycerol. Food Chem..

[80] Fu X.Z., Wang D., Belwal T., Xu Y.Q., Li L., Luo Z.S. (2021). Sonication-synergistic natural deep eutectic solvent as a green and efficient approach for extraction of phenolic compounds from peels of Carya cathayensis Sarg. Food Chem..

[81] Cherif M.M., Grigorakis S., Halahlah A., Loupassaki S., Makris D.P. (2020). High-efficiency extraction of phenolics from wheat waste biomass (Bran) by combining deep eutectic solvent, ultrasound-assisted pretreatment and thermal treatment. Environ. Process..

[82] Huang Y., Feng F., Jiang J., Qiao Y., Wu T., Voglmeir J., Chen Z.G. (2017). Green and efficient extraction of rutin from tartary buckwheat hull by using natural deep eutectic solvents. Food Chem..

[83] Bubalo M.C., Vidovic S., Redovnikovic I.R., Jokic S. (2018). New perspective in extraction of plant biologically active compounds by green solvents. Food Bioprod. Process..

[84] Bakirtzi C., Triantafyllidou K., Makris D.P. (2016). Novel lactic acid-based natural deep eutectic solvents: Efficiency in the ultrasound-assisted extraction of antioxidant polyphenols from common native Greek medicinal plants. J. Appl. Res. Med. Aromat. Plants.

[85] Peng X., Duan M.H., Yao X.H., Zhang Y.H., Zhao C.J., Zu Y.G., Fu Y.J. (2016). Green extraction of five target phenolic acids from Lonicerae japonicae Flos with deep eutectic solvent. Sep. Purif. Technol..

[86] Gao M.Z., Cui Q., Wang L.T., Meng Y., Yu L., Li Y.Y., Fu Y.J. (2020). A green and integrated strategy for enhanced phenolic compounds extraction from mulberry (*Morus alba* L.) leaves by deep eutectic solvent. Microchem. J..

[87] Park H.E., Tang B., Row K.H. (2014). Application of deep eutectic solvents as additives in ultrasonic extraction of two phenolic acids from herba artemisiae scopariae. Anal. Lett..

[88] Liu Y.J., Zhang H., Yu H.M., Guo S.H., Chen D.W. (2019). Deep eutectic solvent as a green solvent for enhanced extraction of narirutin, naringin, hesperidin and neohesperidin from Aurantii Fructus. Phytochem. Anal..

[89] Oktaviyanti N.D., Kartini, Mun’im A. (2019). Application and optimization of ultrasound-assisted deep eutectic solvent for the extraction of new skin-lightening cosmetic materials from Ixora javanica flower. Heliyon.

[90] Pontes P.V.D., Shiwaku I.A., Maximo G.J., Batista E.A.C. (2021). Choline chloride-based deep eutectic solvents as potential solvent for extraction of phenolic compounds from olive leaves: Extraction optimization and solvent characterization. Food Chem..

[91] Mogaddam M.R.A., Farajzadeh M.A., Tuzen M., Jouyban A., Khandaghi J. (2021). Organic solvent-free elevated temperature liquid-liquid extraction combined with a new switchable deep eutectic solvent-based dispersive liquid-liquid microextraction of three phenolic antioxidants from oil samples. Microchem. J..

[92] Li M.Y., Liu Y.Z., Hu F.J., Ren H.W., Duan E.H. (2021). Amino Acid-based natural deep eutectic solvents for extraction of phenolic compounds from aqueous environments. Processes.

[93] Liu L.L., Tang W.Y., Tang B.K., Han D.D., Row K.H., Zhu T. (2017). Pipette-tip solid-phase extraction based on deep eutectic solvent modified graphene for the determination of sulfamerazine in river water. J. Sep. Sci..

[94] Dogan B., Elik A., Altunay N. (2020). Determination of paracetamol in synthetic urea and pharmaceutical samples by shaker-assisted deep eutectic solvent microextraction and spectrophotometry. Microchem. J..

[95] Li G.Z., Zhu T., Row K.H. (2017). Deep eutectic solvents for the purification of chloromycetin and thiamphenicol from milk. J. Sep. Sci..

[96] Jouyban A., Farajzadeh M.A., Khodadadeian F., Khoubnasabjafari M., Mogaddam M.R.A. (2021). Development of a deep eutectic solvent-based ultrasound-assisted homogenous liquid-liquid microextraction method for simultaneous extraction of daclatasvir and sofosbuvir from urine samples. J. Pharm. Biomed..

[97] Fan Y.C., Luo H., Zhu C.Y., Li W.J., Wu D., Wu H.W. (2021). Hydrophobic natural alcohols based deep eutectic solvents: Effective solvents for the extraction of quinine. Sep. Purif. Technol..

[98] Kurtulbas E., Pekel A.G., Toprakci I., Ozcelik G., Bilgin M., Sahin S. (2022). Hydrophobic carboxylic acid based deep eutectic solvent for the removal of diclofenac. Biomass Convers. Biorefin..

[99] Shahi M., Javadi A., Mogaddam M.R.A., Mirzaei H., Nemati M. (2021). Preparation of multiwall carbon nanotube/urea-formaldehyde nanocomposite as a new sorbent in solid-phase extraction and its combination with deep eutectic solvent-based dispersive liquid-liquid microextraction for extraction of antibiotic residues in honey. J. Sep. Sci..

[100] Hadi N.A., Ng M.H., Choo Y.M., Hashim M.A., Jayakumar N.S. (2015). Performance of choline-based deep eutectic solvents in the extraction of tocols from crude palm oil. J. Am. Oil Chem. Soc..

[101] Mohammadi B., Shekaari H., Zafarani-Moattar M.T. (2021). Selective separation of α-tocopherol using eco-friendly choline chloride—Based deep eutectic solvents (DESs) via liquid-liquid extraction. Colloids Surf..

[102] Liu W., Fu X.L., Li Z.Z. (2019). Extraction of tocopherol from soybean oil deodorizer distillate by deep eutectic solvents. J. Oleo Sci..

[103] Ge D.D., Wang Y., Jiang Q., Dai E.R. (2019). A deep eutectic solvent as an extraction solvent to separate and preconcentrate parabens in water samples using in situ liquid-liquid microextraction. J. Braz. Chem. Soc..

[104] Hashemi B., Zohrabi P., Dehdashtian S. (2018). Application of green solvents as sorbent modifiers in sorptive-based extraction techniques for extraction of environmental pollutants. Trends Anal. Chem..

[105] Shakirova F., Shishov A., Bulatov A. (2022). Hydrolysis of triglycerides in milk to provide fatty acids as precursors in the formation of deep eutectic solvent for extraction of polycyclic aromatic hydrocarbons. Talanta.

[106] Nie J., Yu G.W., Song Z.Y., Wang X.J., Li Z.G., She Y.B., Lee M. (2017). Microwave-assisted deep eutectic solvent extraction coupled with headspace solid-phase microextraction followed by GC-MS for the analysis of volatile compounds from tobacco. Anal. Methods.

[107] Zhang L.J., Wang M.S. (2017). Optimization of deep eutectic solvent-based ultrasound-assisted extraction of polysaccharides from Dioscorea opposita Thunb. Int. J. Biol. Macromol..

[108] Shang X.C., Chu D.P., Zhang J.X., Zheng Y.F., Li Y.Q. (2021). Microwave-assisted extraction, partial purification and biological activity in vitro of polysaccharides from bladder-wrack (*Fucus vesiculosus*) by using deep eutectic solvents. Sep. Purif. Technol..

[109] Nie J.G., Chen D.T., Lu Y.B. (2020). Deep eutectic solvents based ultrasonic extraction of polysaccharides from edible brown seaweed sargassum horneri. J. Mar. Sci. Eng..

[110] Das A.K., Sharma M., Mondal D., Prasad K. (2016). Deep eutectic solvents as efficient solvent system for the extraction of kappa-carrageenan from Kappaphycus alvarezii. Carbohydr. Polym..

[111] Liu Y.H., Li J., Fu R.Z., Zhang L.L., Wang D.Z., Wang S. (2019). Enhanced extraction of natural pigments from *Curcuma longa* L. using natural deep eutectic solvents. Ind. Crops Prod..

[112] Patil S.S., Pathak A., Rathod V.K. (2021). Optimization and kinetic study of ultrasound assisted deep eutectic solvent based extraction: A greener route for extraction of curcuminoids from Curcuma longa. Ultrason. Sonochem..

[113] Aydin F., Yilmaz E., Soylak M. (2018). Vortex assisted deep eutectic solvent (DES)-emulsification liquid-liquid microextraction of trace curcumin in food and herbal tea samples. Food Chem..

[114] Zhang H., Tang B., Row K.H. (2014). A green deep eutectic solvent-based ultrasound-assisted method to extract astaxanthin from shrimp byproducts. Anal. Lett..

[115] Wang W.D., Du Y.G., Xiao Z.E., Li Y., Li B.F., Yang G.W. (2017). Determination of trace rhodamine B in chili oil by deep eutectic solvent extraction and an ultra high-performance liquid chromatograph equipped with a fluorescence detector. Anal. Sci..

[116] Su E.Z., Yang M., Cao J., Lu C., Wang J.H., Cao F.L. (2017). Deep eutectic solvents as green media for efficient extraction of terpene trilactones from Ginkgo biloba leaves. J. Liq. Chromatogr. Relat. Technol..

[117] Ge D.D., Zhang Y., Dai Y.X., Yang S.M. (2018). Air-assisted dispersive liquid-liquid microextraction based on a new hydrophobic deep eutectic solvent for the preconcentration of benzophenone-type UV filters from aqueous samples. J. Sep. Sci..

[118] Rabhi F., Di Pietro T., Mutelet F., Sifaoui H. (2021). Extraction of butanol and acetonitrile from aqueous solution using carboxylic acid based deep eutectic solvents. J. Mol. Liq..

[119] Wang M., Wang J.Q., Zhang Y., Xia Q., Bi W.T., Yang X.D., Chen D.D.Y. (2016). Fast environment-friendly ball mill-assisted deep eutectic solvent-based extraction of natural products. J. Chromatogr. A.

[120] Zhang H., Tang B., Row K. (2014). Extraction of catechin compounds from green tea with a new green solvent. Chem. Res. Chin. Univ..

[121] Zhao Y.P., Wang P., Zheng W., Yu G.W., Li Z.G., She Y.B., Lee M. (2019). Three-stage microwave extraction of cumin (*Cuminum cyminum* L.) Seed essential oil with natural deep eutectic solvents. Ind. Crops Prod..

[122] Xu F.-X., Zhang J.-Y., Jin J., Li Z.-G., She Y.-B., Lee M.-R. (2021). Microwave-assisted natural deep eutectic solvents pretreatment followed by hydrodistillation coupled with GC-MS for analysis of essential oil from turmeric (*Curcuma longa* L.). J. Oleo Sci..

[123] Milani G., Vian M., Cavalluzzi M.M., Franchini C., Corbo F., Lentini G., Chemat F. (2020). Ultrasound and deep eutectic solvents: An efficient combination to tune the mechanism of steviol glycosides extraction. Ultrason. Sonochem..

